# Association of varicose veins with incidence risk of atrial fibrillation: a population-based cohort study

**DOI:** 10.1097/JS9.0000000000002036

**Published:** 2024-08-14

**Authors:** Soyoun Choi, Gwang-Hyun Leem, Tae-Jin Song

**Affiliations:** a Department of Neurology, Seoul Hospital, Ewha Womans University College of Medicine; b Medical Research Institute, Ewha Womans University College of Medicine, Seoul, Republic of Korea

**Keywords:** atrial fibrillation, chronic venous disease, varicose vein

## Abstract

**Background:**

Varicose veins (VV) were once considered benign and common ailments; however, recent research suggests a potential link between VV and cardiovascular diseases or mortality. VV share common risk factors and pathophysiology with cardiovascular disease, potentially influencing the vascular system. Therefore, the authors aimed to investigate the association between VV and the incidence risk of atrial fibrillation (AF) using a population-based cohort.

**Methods:**

Our retrospective cohort study included 2 680 971 individuals who underwent examination through the Korean National Health Screening Service from 2010 to 2011. VV was defined by two or more claims with the International Classification of Diseases 10th Revision diagnostic codes: I83.0, I83.1, I83.2 (VV of lower extremities with ulcer or inflammation, severe VV), and I83.9 (asymptomatic VV of lower extremities, mild VV). The 1:3 propensity score matching (PSM) was used to assess the risk of newly developed AF, identified via insurance claims coded as I48.

**Results:**

The mean age of all participants was 48.5±14.2 years, with 51.4% being male. Among the population, 24 557 (0.91%) had VV, including 3684 (0.14%) of severe VV and 20 873 (0.77%) of mild VV. During a median follow-up of 10.06 years, 24 557 (0.92%) cases of AF occurred. Participants with VV exhibited an increased incidence risk of AF compared to those without it before (HR: 1.13, 95% CI: 1.06–1.21, *P*<0.001) and after PSM (HR: 1.17, 95% CI: 1.08–1.27, *P*<0.001). This positive association was consistently observed in severe VV both before (HR: 1.19, 95% CI [1.09–1.28], *P*=0.002) and after PSM (HR: 1.20, 95% CI [1.10–1.30], *P*=0.003) and mild VV also before (HR: 1.10, 95% CI [1.04–1.16], *P*=0.003) and after PSM (HR: 1.13, 95% CI [1.03–1.–20], *P*<0.001).

**Conclusions:**

These findings suggest that VV may be associated with an increased risk of AF. Hence, the presence of VV should be considered as an association factor for AF occurrence.

## Graphical abstract

**Figure:**
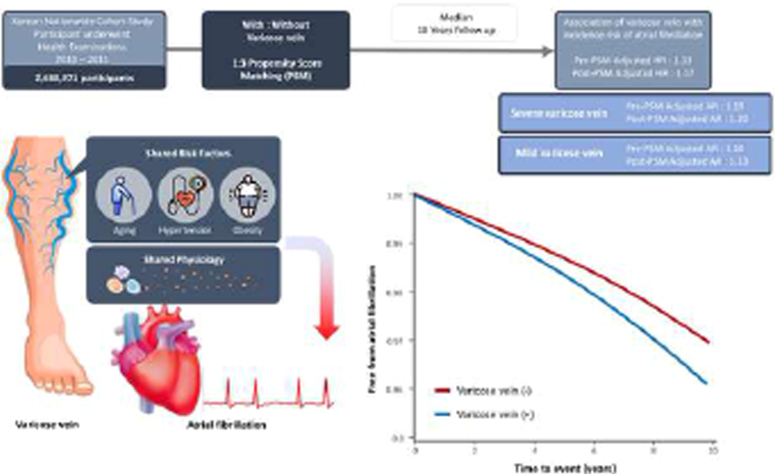

## Introduction

Highlights

- Varicose veins, a common manifestation of chronic venous insufficiency, are associated with cardiovascular disease, but there is little data on their association with atrial fibrillation.
- Our population-based cohort study suggests a potential association between the presence of varicose veins and an increased incidence risk of atrial fibrillation.
- Varicose veins could be considered as an association factor for atrial fibrillation occurrence.

Varicose veins (VV) are superficial veins that become enlarged and twisted, typically with a diameter of 3 mm or more. These veins are located beneath the skin and primarily impact the saphenous veins, their tributaries, or other superficial veins in the legs that are not part of the saphenous system^1^. Several factors increase the risk of developing VV, including being female, having multiple childbirths, being overweight, and experiencing chronic constipation^2^. Additionally, prolonged periods of standing or walking in a work environment can contribute to its onset^3^.

Atrial fibrillation (AF) is among the most prevalent types of heart rhythm disorders globally, which is closely associated with severe health complications, including the risk of systemic thromboembolism^4–7^. This condition significantly elevates the risks of mortality and stroke, highlighting its status as a major health concern^8^. AF prevalence is increasing, driven by an aging population and increasing incidence of heart and brain-related diseases^9–12^. Consequently, identifying and understanding factors contributing to the risk of developing AF is paramount. Known contributors include hypertension, cardiac muscle diseases, tobacco use, and alcohol consumption. However, studies on identifying and addressing modifiable risk factors that can influence AF development are urgently needed^13,14^.

While VV is a common clinical manifestation of chronic venous disease, its association with various diseases has frequently been underestimated. Recent findings indicate that chronic venous insufficiency is independently associated with an increased risk of cardiovascular^15,16^ and peripheral artery diseases^17^. This association is more pronounced with higher Clinical-Etiology-Anatomy-Pathophysiology (CEAP) stages^15^. This association may stem from shared risk factors such as hypertension, diabetes, obesity, smoking, or shared pathophysiology involving endothelial dysfunction^3,18^. Nevertheless, research on the connection between chronic venous disease and the incidence risk of AF remains limited. We hypothesized that the presence of VV could be associated with an increased risk of AF. Therefore, this study aimed to examine the association between the presence and treatment of VV and the incidence risk of AF in a population-based longitudinal study.

## Methods

### Data source

The South Korean National Health Insurance System (NHIS) offers a comprehensive database that encompasses demographic details, socioeconomic background, medical diagnoses, and treatment information. Additionally, it supports a national health examination database and a database for medical care facilities^19^. The NHIS policy recommends that its members undergo standardized health check-ups every 2 years^20^. In this study, data from the NHIS-National Health Screening Cohort (NHIS-HEALS)^21,22^ were utilized, which comprised individuals who participated in the NHIS medical health screening programs. Our dataset comprised a randomly selected 15% sample of the general Korean population aged ≥40 years who underwent the NHIS-National Health Screening Examination. From this cohort, we collected data on demographic information, height, weight, household income, smoking and alcohol habits, physical activity levels, and existing medical history and health conditions. The research was approved by the Institutional Review Board of a university hospital in Seoul (Approval number: 2024-03-006).

### Study population and variables

**Figure 1:**
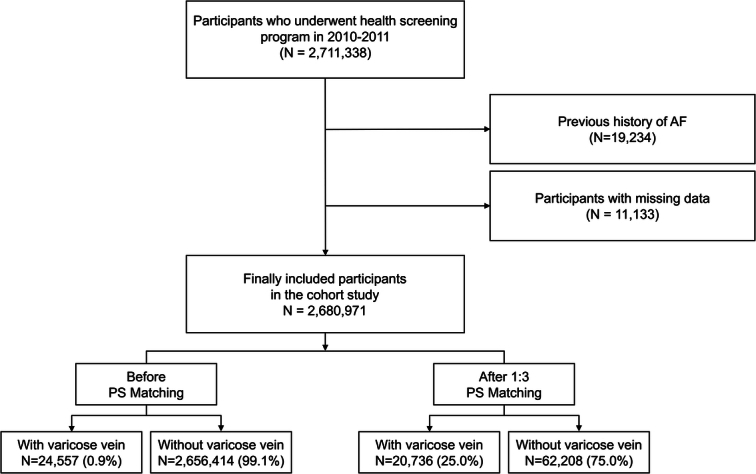
Flowchart of inclusion and exclusion criteria.

Participants who underwent health examinations between 2010 and 2011 (*n*=2 711 338) were included in this study. However, those with missing data for at least one of the variables used in this study (*n*=11 133) and a history of AF (*n*=19 234) were excluded. Finally, 2 680 971 participants were included (Fig. 1).

### Definition of varicose veins and covariates

The presence of VV was defined as having two or more claims with International Classification of Diseases 10th Revision (ICD-10) diagnostic codes: I83.0 (VV of lower extremities with ulcer), I83.1 (VV of lower extremities with inflammation), I83.2 (VV of lower extremities with ulcer and inflammation), and I83. 9 (asymptomatic VV of lower extremities)^23^. In the CEAP classification, clinical signs of chronic venous disorders are graded based on severity from C0 to C6, indicating no visible signs and active venous ulcers, respectively^3^. Moreover, the CEAP classification categorizes the pathophysiology of chronic venous disease into reflux, obstruction, or a combination of both^3^. Venous reflux results from valve incompetence, inflammation, and venous hypertension, which exacerbate each other and ultimately contribute to disease progress, while obstruction is typically due to thrombosis^24^. When applying the CEAP classification to VV based on ICD-10 codes (I83.0, I83.1, I83.2, and I83.9), the presence of VV can be classified as C2 or higher. Moreover, ICD-10 codes I83.0, I83.1, and I83.2 can be classified as C5 or higher, while ICD-10 codes I83.9 can be classified between C2 and C4^3,23^. This definition was based on a previous study that examined the national trends in treating chronic venous diseases, including VV^25^.

To investigate the treatment effect of VV, we assessed whether participants had undergone surgical treatment for VV. The definition of treatment for VV was established based on two criteria: the presence of the above-mentioned diagnostic codes for VV and the matching of appropriate surgical treatment codes. Eligible surgical treatments covered by health claims include saphenous vein ligation and stab avulsion + perforator ligation (O0261), saphenous vein ligation and stab avulsion - perforator ligation (O0262), segmental stripping and stab avulsion + perforator ligation (O0263), segmental stripping and stab avulsion - perforator ligation (O0264), total stripping and stab avulsion + perforator ligation (O0265), total stripping and stab avulsion - perforator ligation (O0266), varicose vein operation, others (perineum) (O0267), local resection (O2052), 1–3 sites(O0215), 4–6 sites (O0216), and >7 sites (O0217))^25^.

As covariates, we investigated age, sex, BMI, household income, smoking status, alcohol consumption, physical activity level, hypertension, diabetes mellitus, dyslipidemia, chronic obstructive pulmonary disease, liver disease, renal disease, stroke, myocardial infarction, and cancer. Detailed definitions for these covariates can be found in the Supplementary Methods^26–29^ (Supplemental Digital Content 1, http://links.lww.com/JS9/D312).

### Outcome

To measure the outcome, the index date was defined as the most recent health examination date. The outcome centered on individuals who had filed one or more insurance claims with the ICD-10 code I48 for AF—a code previously validated (with a positive predictive value of 94.1%)^30,31^. In this study, individuals who had a prior diagnosis of AF or had filed claims related to AF treatment before the index date were not considered as AF incidences. Therefore, in our study, the definition of AF incidence referred to hospitalized patients who made their first claim for AF treatment after the index date. Follow-up was conducted until 31 December 2020, death, or the first AF incidence.

### Statistical analysis

Baseline characteristics between the group with VV and those without were compared using the independent *t*-test for continuous variables and the *χ* ^2^ test (or Fisher’s exact test) for categorical variables. To balance baseline characteristics and control for potential confounders between both groups, we used 1:3 propensity score matching (PSM)^32,33^. The adequacy of PSM was assessed using the standardized mean difference (SMD), and PSM was considered appropriate when the absolute values of SMD were <0.1^34,35^.

To analyze the incidence risk of AF, we utilized Kaplan–Meier survival curves and examined group differences with log-rank tests. Additionally, we employed Cox proportional hazard models to calculate hazard ratios (HR) and 95% CI. We conducted subgroup analyses to assess the association between the presence of VV and AF across demographic data and covariates, assessing interactions with *P*-value for interaction and visualizing the results using Forest plots. To assess the severity of VV, we performed sensitivity analysis based on ICD-10 codes: I83.0, I83.1, and I83.2 for severe VV and I83.9 for mild VV.

To minimize reserve causality, landmark analysis was performed where the outcome was defined as the occurrence of AF 1 year after the index date. For subgroup analysis, we evaluated the incidence risk of AF based on whether a surgical treatment for VV was performed or not, using Cox regression analysis. All statistical analyses were performed using SAS 9.4 version (SAS Inc.) and R software, version 4.2.1 (R Foundation for Statistical Computing). We considered statistical significance for all tests as a two-sided *P*-value <0.05.

This study was reported in accordance with the strengthening the reporting of cohort, cross-sectional, and case–control studies in surgery (STROCSS) criteria^36^ (Supplemental Digital Content 2, http://links.lww.com/JS9/D313).

## Results

Table 1 shows the baseline characteristics according to the presence of VV. The mean age of all participants was 48.5±14.2 years, with 51.4% being male. Among the included population of 2 680 971, 24 557 (0.91%) individuals had VV with corresponding ICD-10 codes of I83.0, I83.1, I83.2, and I83.9. Considering the severity of VV, 3684 (0.14%) individuals had ICD-10 codes of I83.0, I83.1, and I83.2 (severe VV), while 20 873 (0.77%) had the ICD-10 code of I83.9 (mild VV). The populations with VV were older individuals and predominantly female. Individuals with VV were less likely to have been current smokers or heavy alcohol consumers. In contrast, the VV population exhibited a higher frequency of covariates, including hypertension, diabetes mellitus, dyslipidemia, chronic obstructive pulmonary disease, liver disease, renal disease, stroke, and cancer (except myocardial infarction) (Table 1). After PSM, the populations with and without VV were well-balanced in terms of matching (Supplementary Table 1, Supplemental Digital Content 3, http://links.lww.com/JS9/D314).

**Table 1:** Baseline characteristics of participants stratified by varicose vein history.

Variable	Total	Varicose vein history		*P*
		No	Yes	
Number of participants (%)	2 680 971	2 656 414 (99.1)	24 557 (0.9)	
Age, years	48.50±14.20	48.47±14.21	52.40±12.56	<0.001
Sex			0	<0.001
Male	1 377 952 (51.4)	1 370 054 (51.6)	7898 (32.2)	
Female	1 303 019 (48.6)	1 286 360 (48.4)	16 659 (67.8)	<0.001
BMI (kg/m^2^)	23.73±3.27	23.73±3.27	23.74±3.18	0.423
Household income				<0.001
Q1, lowest	531 151 (19.8)	526 210 (19.8)	4941 (20.1)	
Q2	570 815 (21.3)	566 026 (21.3)	4789 (19.5)	
Q3	718 155 (26.8)	711 965 (26.8)	6190 (25.2)	
Q4, highest	860 850 (32.1)	852 213 (32.1)	8637 (35.2)	
Smoking status				<0.001
Never	1 652 197 (61.6)	1 633 472 (61.5)	18 725 (76.2)	
Former	387 565 (14.5)	384 674 (14.5)	2891 (11.8)	
Current	641 209 (23.9)	638 268 (24.0)	2941 (12.0)	
Alcohol consumption (days/week)				<0.001
None	1 425 416 (53.2)	1 409 396 (53.1)	16 020 (65.2)	
1-2	891 028 (33.2)	884 880 (33.3)	6148 (25.0)	
3-4	257 112 (9.6)	255 503 (9.6)	1609 (6.6)	
≥5	107 415 (4.0)	106 635 (4.0)	780 (3.2)	
Regular physical activity (days/week)				<0.001
None	1 654 507 (61.7)	1 639 119 (61.7)	15 388 (62.7)	
1-2	620 952 (23.2)	616 222 (23.2)	4730 (19.3)	
3-4	251 431 (9.4)	248 817 (9.4)	2614 (10.6)	
≥5	154 081 (5.8)	152 256 (5.7)	1825 (7.4)	
Comorbidities				
Hypertension	616 635 (23)	609 192 (22.9)	7443 (30.3)	<0.001
Diabetes mellitus	250 363 (9.3)	247 473 (9.3)	2890 (11.8)	<0.001
Dyslipidemia	433 856 (16.2)	427 498 (16.1)	6358 (25.9)	<0.001
Chronic obstructive pulmonary disease	172 917 (6.5)	170 562 (6.4)	2355 (9.6)	<0.001
Liver disease	153 991 (5.7)	151 811 (5.7)	2180 (8.9)	<0.001
Renal disease	32 172 (1.2)	34 533 (1.3)	712 (2.9)	<0.001
Stroke	43 601 (1.6)	43 070 (1.6)	531 (2.2)	<0.001
Myocardial infarction	12 592 (0.4)	12 486 (0.4)	106 (0.4)	0.536
Cancer	46 748 (1.7)	46 040 (1.7)	708 (2.9)	<0.001

*P-*value by *χ* ^2^ test. Data are expressed as the mean±SD, or *n* (%).

Q, quartile.

**Figure 2:**
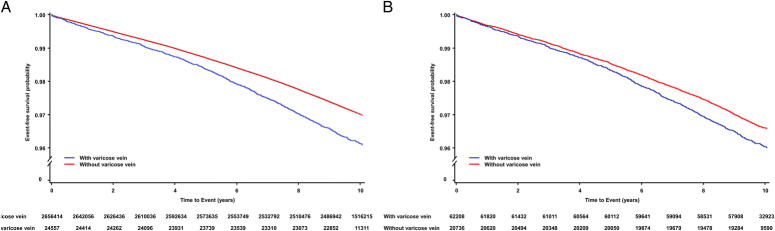
Kaplan–Meier survival curves for atrial fibrillation based on the presence of varicose vein (A) before and (B) after PSM.

During a median follow-up of 10.06 years (interquartile range 9.43–10.44), 24 557 (0.92%) cases of AF occurred. Among these, 24 115 (98.2%) cases were categorized as nonvalvular AF, while 442 (1.8%) cases were observed as valvular AF. Kaplan–Meier survival curves showed that AF occurrence was dependent on the presence of VV before and after PSM (*P*<0.001) (Fig. 2A, B). In the multivariate analysis, the group with VV consistently showed an increased incidence risk of AF than the group without it, both before (HR: 1.13, 95% CI [1.06–1.21], *P*<0.001) and after PSM (HR: 1.17, 95% CI [1.08–1.27], *P*<0.001) (Table 2, Supplementary Table 2, Supplemental Digital Content 3, http://links.lww.com/JS9/D314 and 3, Supplemental Digital Content 3, http://links.lww.com/JS9/D314).

**Table 2:** Association of the presence of varicose vein with incidence risk of atrial fibrillation.

	Number of participants	Number of events	Event rate (%) (95% CI)	Person-years	Incidence rate (Per 1000 person-years)	Adjusted HR (95% CI)	*P*
Before-PSM Presence of varicose vein history							
(−)	2 656 414	78 061	2.94 (2.92–2.96)	25 667 331.99	3.04	1 (reference)	
(+) I83.0, I83.1, I83.2, I83.9	24 557	925	3.77 (3.53–4.00)	234 049.96	3.95	1.13 (1.06–1.21)	<0.001
(+) I83.0, I83.1, I83.2	3684	163	4.42 (4.31–4.72)	35 982.35	4.53	1.19 (1.09–1.28)	0.002
(+) I83.9	20 873	762	3.65 (3.32–3.98)	201 587.30	3.78	1.10 (1.04–1.16)	0.003
After PSM Presence of varicose vein history							
(−)	62 208	2072	3.33 (3.19–3.47)	598 125.98	3.46	1 (reference)	
(+) I83.0, I83.1, I83.2, I83.9	20 736	802	3.87 (3.61–4.13)	197 665.48	4.06	1.17 (1.08–1.27)	<0.001
(+) I83.0, I83.1, I83.2	3110	136	4.38 (4.12–4.63)	30 846.21	4.42	1.18 (1.10–1.26)	0.003
(+) I83.9	17 626	666	3.76 (3.42–4.01)	173 801.56	3.83	1.08 (1.03–1.13)	<0.001

The multivariable model was adjusted with age, sex, BMI, income levels, smoking, alcohol consumption, regular physical activity, hypertension, diabetes mellitus, dyslipidemia, chronic obstructive pulmonary disease, liver disease, renal disease, stroke, myocardial infarction, and cancer.

HR, hazard ratio; PSM, propensity score matching.

**Figure 3:**
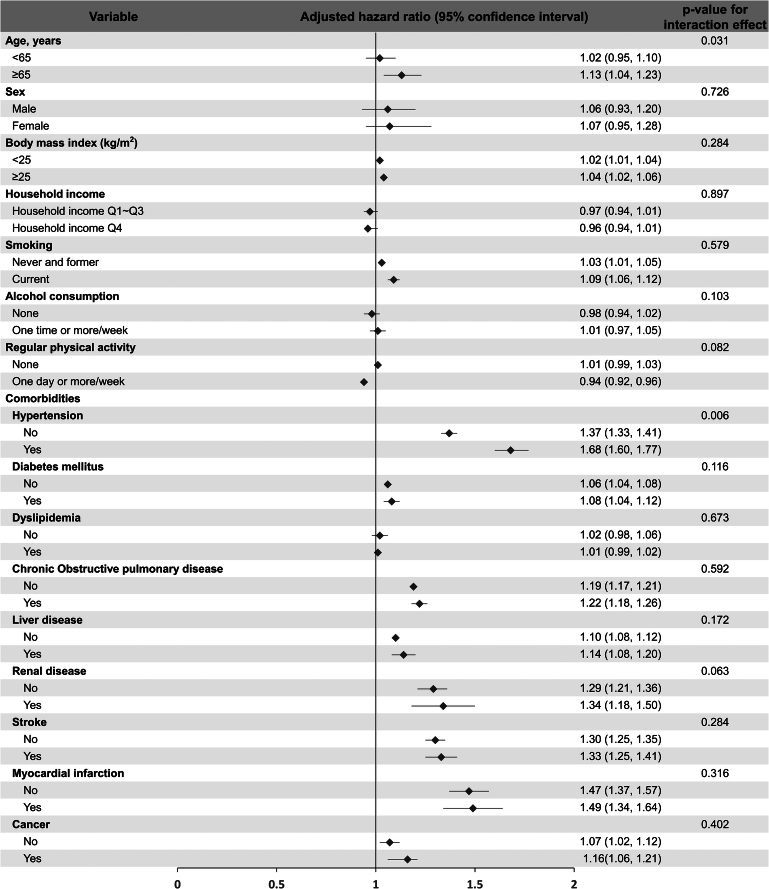
Forest plots depicting the association of varicose vein with atrial fibrillation according to covariate. PSM, propensity score matching; Q, quartile.

In subgroup analysis, the association between the presence of VV and the incidence risk of AF was consistently observed regardless of covariates. Furthermore, the elderly population (HR: 1.02, 95% CI [0.95–1.10] for <65 years and HR: 1.13, 95% CI [1.04–1.23] for ≥65 years, *P* for interaction=0.031) and those with a history of hypertension (HR: 1.37, 95% CI [1.33–1.41] for without hypertension and HR: 1.68, 95% CI [1.60–1.77] for with hypertension, *P* for interaction=0.006) showed significantly higher incidence risk of AF (Fig. 3, Supplementary Table 4, Supplemental Digital Content 3, http://links.lww.com/JS9/D314).

In sensitivity analysis, the group with ICD-10 codes of I83.0, I83.1, and I83.2 (indicating severe VV, CEAP grade ≥5) consistently demonstrated an increased incidence risk of AF than the group without it, both before (HR: 1.19, 95% CI [1.09–1.28], *P*=0.002) and after PSM (HR: 1.20, 95% CI [1.10–1.30], *P*=0.003). Moreover, the group with ICD-10 codes of I83.9 (indicating mild VV, CEAP grade 2 to 4) consistently showed an increased incidence risk of AF than the group without VV, both before (HR: 1.10, 95% CI [1.04–1.16], *P*=0.003) and after PSM (HR: 1.13, 95% CI [1.03–1.20], *P*<0.001) (Table 2, Supplementary Table 5, Supplemental Digital Content 3, http://links.lww.com/JS9/D314 and 6, Supplemental Digital Content 3, http://links.lww.com/JS9/D314). In the landmark analysis, the relationship between the presence of VV and incidence risk of AF was also consistently demonstrated (Supplementary Tables 7–9, Supplemental Digital Content 3, http://links.lww.com/JS9/D314).

Supplementary Table 10 (Supplemental Digital Content 3, http://links.lww.com/JS9/D314) presents a descriptive analysis of the detailed frequency of surgical treatment for VV. Regarding the association with the treatment of VV, the population that underwent surgical treatment for VV showed a nonsignificantly lower risk of AF incidence than those who did not undergo surgical treatment (HR: 0.96, 95% CI [0.91–1.01], *P*=0.092). This trend was observed regardless of subgroup, including those with ICD-10 codes of I83.0, I83.1, and I83.2 (HR: 0.95, 95% CI [0.90–1.00], *P*=0.069) and those with ICD-10 codes of I83.9 (HR: 0.97, 95% CI [0.93–1.01], *P*=0.098) (Table 3, Supplementary Table 11, Supplemental Digital Content 3, http://links.lww.com/JS9/D314).

**Table 3:** Association of the surgical treatment of varicose vein with incidence risk of atrial fibrillation.

Surgical treatment	Number of participants	Number of events	Event rate (%) (95% CI)	Person-years	Incidence rate (Per 1000 person-years)	Adjusted HR (95% CI)	*P*
No	15 907	607	3.82 (3.52–4.11)	150 993.02	4.02	1 (reference)	
Yes							
I83.0, I83.1, I83.2, I83.9	8650	318	3.68 (3.28–4.07)	83 056.94	3.83	0.96 (0.91–1.01)	0.092
I83.0, I83.1, I83.2	8026	295	3.67 (3.26–4.09)	77 641.78	3.80	0.95 (0.90–1.00)	0.069
I83.9	624	23	3.68 (3.26–4.10)	5989.54	3.84	0.97 (0.93–1.01)	0.098

The multivariable model was adjusted with age, sex, BMI, income levels, smoking, alcohol consumption, regular physical activity, hypertension, diabetes mellitus, dyslipidemia, chronic obstructive pulmonary disease, liver disease, renal disease, stroke, myocardial infarction, and cancer.

HR, hazard ratio.

## Discussion

The key findings of our study are as follows: First, participants with VV had a higher incidence risk of AF than those without it, and this association remained consistent even after PSM. Second, our subgroup analysis revealed that the risk association between VV and AF persisted across various covariates, with notable significance in the elderly population and those with a history of hypertension.

Research examining the relationship between VV and an increased incidence of AF is scarce. A single study was published in 2022 under Taiwan’s national health insurance research program^37^. The research from Taiwan, an age- and sex-matched population-based study, indicates that individuals with VV had an adjusted HR of 1.23 for AF^37^.

Our study has several strengths, including a significantly larger sample size—more than double that of the previous study—and the application of PSM in a general population, which demonstrates the association between VV and an elevated incidence of AF.

The association between VV and AF is assessed from three perspectives: shared risk factors, shared pathophysiology, or the bridging role of certain diseases or conditions. Shared risk factors for VV and AF include age, diabetes, arterial hypertension, obesity, dyslipidemia, and smoking^13,15,38–40^. Consequently, individuals exposed to these similar risk factors are at an increased risk of developing both conditions.

Another perspective involves shared pathophysiology. In VV, the pathophysiology is characterized by the complex interaction between structural changes in the vascular wall and inflammatory responses^41,42^. The normal venous wall comprises the intima, media, and adventitia. In VV, the most critical change involves hypertrophy of the intimal endothelial cell layer^41^, which disrupts the balance of collagen and elastin and impairs smooth muscle cell function. This leads to compromised venous elasticity, blood reflux, venous dilation, and valve dysfunction^41^. Inflammatory responses also significantly contribute to VV progression, involving chronic inflammation characterized by leukocyte accumulation and increased proinflammatory cytokines^43^. Matrix metalloproteinases (MMPs) contribute to varicose vein formation by degrading the extracellular matrix, thereby weakening and dilating the venous wall^44^. Additionally, the action of MMPs increases and dysregulated apoptosis leads to decreased smooth muscle cell turnover, which weakens venous walls and vein dilation^45,46^. MMPs are closely linked to myocardial remodeling, potentially contributing to AF^47^. A study demonstrated elevated levels of systemic inflammatory substances such as C-reactive protein (CRP) and interleukin 6 in blood extracted from VV compared to systemic blood from one person^48^. The pathophysiology of AF also involves systemic inflammation. Studies demonstrate elevated levels of CRP in individuals with AF, suggesting systemic inflammation as a potential shared pathophysiological mechanism^49,50^.

The third perspective considers the presence of intermediate diseases or conditions that might mediate the relationship between VV and AF. Conditions such as peripheral artery or cardiovascular disease could potentially serve as intermediaries. Chronic venous disease increases systemic inflammation biomarkers, potentially leading to endothelial dysfunction and contributing to major adverse cardiovascular events^51^. Several studies also suggest that VV may elevate the incidence of cardiovascular diseases^15–17,52^. Additionally, a study indicated an association between VV and coronary artery ectasia, suggesting that vascular wall defect leading to overproduction of MMPs could result in vessel wall dilation^53^. Our study findings enhance our understanding of the association between VV and AF, underscoring shared risk factors and pathophysiological conditions.

In subgroup analysis, a significant significance between VV and AF was observed among elderly individuals and those with a history of hypertension. Age has consistently emerged as a robust risk factor for VV and AF across various studies^38–40^. Arterial hypertension has been widely reported as a risk factor for AF^13,38^; however, its association with VV has been less frequently discussed^15^. Consequently, a population with VV, especially when accompanied by aging or hypertension, may potentially face an increased risk for future AF incidence. Furthermore, generalizing our findings for application across all age groups may be challenging, given the lack of a significant association between the presence of VV and the incidence risk of AF in relatively young individuals.

Studies examining whether surgical treatment or interventions of VV can reduce the risk of AF remain few. A previous study shows that endovenous thermal ablation therapy in patients with VV is associated with reduced incidence rates of deep vein thrombosis and peripheral artery disease^54^. Nevertheless, our findings indicate that neither surgical procedures nor treatments for VV attenuated the incident risk of AF. In our study, we could not establish the cause of the association between VV and AF, as even treatment for VV did not alter the prevalence of AF. Moreover, other important surgical treatment strategies for VV, including radiofrequency endovenous closure, transilluminated powered phlebectomy, and endovenous laser treatment, are not covered by health insurance. Therefore, even if these procedures were performed, they could not be included in our dataset. This discrepancy could lead to underestimating the number of individuals who actually underwent surgical treatment. Furthermore, it may explain why surgical treatment of VV did not show a significant association with the incidence risk of AF in our results. Therefore, further study is warranted to determine whether surgical treatment of VV can mitigate AF risk.

Our study has some limitations. First, our results may be subjected to ethnic bias, potentially limiting the generalizability of our conclusions across different demographic groups. Therefore, conducting additional research that encompasses diverse racial and ethnic populations is crucial. Second, relying on insurance claims data presented several challenges: establishing a biological plausibility between chronic venous insufficiency and AF was not possible owing to the lack of evidence for the underlying mechanism, determining the etiology of venous insufficiency for each participant, or providing a more detailed CEAP classification. Particularly for C2-C4, accurate determination of the presence or absence of edema, pigmentation, venous eczema, or lipodermatosclerosis was challenging. In addition, the classification was limited by constraints in establishing etiology, anatomical, and pathophysiologic aspects. Third, although our dataset was adjusted for important covariates and PSM was performed, potential confounding factors such as venous thromboembolism were not be included. Finally, as a retrospective population-based cohort study, our research encountered inherent challenges in tracking the progression of VV and establishing clear cause-and-effect relationships.

## Conclusions

Our study suggests a potential association between VV and an increased incidence risk of AF. Hence, the presence of VV should be considered as an association factor for AF occurrence. However, further research is necessary to elucidate the potential connections between VV and AF.

## Ethical approval

Ethical approval for this study was obtained from the Institutional Review Board of Ewha Womans University Seoul Hospital (SEUMC 2024-03-006).

## Consent

This study used anonymized data from the South Korean National Health Insurance System (NHIS), with ethical approval granted by the Institutional Review Board of Ewha Womans University Seoul Hospital (Approval Number: SEUMC 2024-03-006). This approval covers all legal and ethical requirements for using NHIS data. All personal identifiers were removed to maintain participant confidentiality and privacy. No personally identifiable information was used in the reporting or publication of this study.

## Source of funding

This work was partly supported by Institute of Information & communications Technology Planning & Evaluation (IITP) grant funded by the Korea government(MSIT) (RS-2022-II220621, Development of artificial intelligence technology that provides dialog-based multi-modal explainability) and Korea Evaluation Institute of Industrial Technology(KEIT) grant funded by the Korea government. This research was supported by a grant from the Korea Health Technology R&D Project through the Korea Health Industry Development Institute (KHIDI), funded by the Ministry of Health & Welfare, Republic of Korea (grant number: RS-2023-00262087 to TJS). The funding source had no role in the design, conduct, or reporting of this study.

## Author contribution

S.C.: writing and data analysis; G.-H.L.: data collection, data analysis, or interpretation; T.-J.S.: study concept, investigation, writing, and supervision.

## Conflicts of interest disclosure

The authors declare that they have no conflicts of interest.

## Research registration unique identifying number (UIN)

1. Name of the registry: not applicable.
2. Unique identifying number or registration ID: not applicable.
3. Hyperlink to your specific registration (must be publicly accessible and will be checked): not applicable.

## Guarantor

Tae-Jin Song, MD, PhD.

## Data availability statement

Data for this analysis were obtained from the NHIS-HEALS database. The availability of these data is restricted and was permitted under a specific license for this study. Requests for NHIS data access can be directed to the National Health Insurance Sharing Service website (http://nhiss.nhis.or.kr/bd/ab/bdaba021eng.do). Access requirements include a completed application form, a detailed research proposal, and an Institutional Review Board approval, all subject to review by the NHIS research support inquiry committee.

## Provenance and peer review

Not commissioned, externally peer-reviewed.

## Supplementary Material
