# Supplementary material for: Association of varicose veins with incidence risk of atrial fibrillation: a population-based cohort study
Source: Int J Surg. 2024 Aug 14;110(9):5704–12. doi: 10.1097/JS9.0000000000002036 (PMC11392101; doi:10.1097/JS9.0000000000002036)
Supplement: Supplementary file 1 [file js9-110-5704-s001.docx]

**Supplementary Methods**

Definition of covariates

Smoking history (never, former, and current)

Definition and measurement of variables like usual smoking habits were obtained by questionnaire in the health examination program. Smoking status was used to categorize participants into three groups: none, former smoker, and current smoker. Current smoker was classified according to the WHO definition as a person who has smoked more than five packs (100 cigarettes) in a lifetime and smoked daily or occasionally for the last 28 days. Former smoker was defined as a person who had smoked more than 100 cigarettes in a lifetime and had not smoked in the last 28 days.

Alcohol consumption (none, moderate, and heavy)

Definition and measurement of variables like usual alcohol consumption were obtained by questionnaire in the health examination program. Alcohol consumption was categorized into three groups: none, moderate drinker, and heavy drinker. Differentiation between moderate and heavy drinker was based on whether a patient usually takes more than 14 drinks/7 drinks per week for men/women. The drinks were calculated by multiplying the average drinking frequency per week by the number of drinks per occasion.

Physical activity (low, moderate)

Physical activity was assessed using the Korean version of the International Physical Activity Questionnaire-short form. We created composite physical activity based on Metabolic Equivalent Task (MET)-minutes/week (walking: 3.3 METs; moderate physical activity: 4.0 METs; vigorous physical activity: 8.0 METs), which was categorized as follows based on total physical activity metabolic equivalents: low (< 600 METs), moderate (600–2,999 METs), and vigorous (≥ 3,000 METs).

Comorbidities

Hypertension

Hypertension was defined as using at least one claim of ICD-10 code(I10–15) with the prescription of an anti-hypertensive agent, claims of ICD-10 code(I10–15) more than two times, a systolic blood pressure of ≥140 mmHg and a diastolic blood pressure of ≥90 mmHg or positive checking in self-report questionnaire on hypertension in the health examination program.

Diabetes mellitus

Diabetes mellitus was as defined using at least one claim of ICD-10 code(E11–14) with the prescription of an anti-diabetic agent, claims of ICD-10 code(E11–14) more than two times, fasting serum glucose concentration of ≥7.0 mmol/L or positive checking in self-report questionnaire on diabetes mellitus in the health examination program.

Dyslipidemia

Dyslipidemia was defined as using at least one claim of ICD-10 code(E78) with the prescription of an anti-dyslipidemic agent, claims of ICD-10 code(E78) more than two times or total cholesterol level of ≥240 mg/dL.

Chronic obstructive pulmonary disease

Chronic obstructive pulmonary disease was defined as using claims of ICD-10 codes (J42, J43 (except J43.0), J44) with more than two times of admission or outpatient department.

Liver disease

Liver disease was defined as using claims of ICD-10 codes (B18, K70, K71, K72, K73, K74, K76.1) with more than two times of admission or outpatient department.

Renal disease

Renal disease was defined as using claims of ICD-10 codes (N17–19, I12–13, E08.2, E10.2, E11.2, E13.2) more than two times or estimated glomerular filtration rate (eGFR) of <60 mL/min/1.73m^2^.

Stroke

Stroke was defined as using claims of ICD-10 codes (I60–I63) with primary diagnosis, admission≥1 times (≥3 days) and brain imaging (CT or MRI) ≥1 times.

Myocardial infarction

Myocardial infarction was defined as using claims of ICD-10 codes (I21, I22) with primary diagnosis with admission of one and more times.

Cancer

Cancer was defined as using claims of ICD-10 code(C00–C97) more than two times with cancer-specific deductible code (V027, V193–4) from the Health Insurance Review and Assessment Service.
