# Supplementary material for: Association of varicose veins with incidence risk of atrial fibrillation: a population-based cohort study
Source: Int J Surg. 2024 Aug 14;110(9):5704–12. doi: 10.1097/JS9.0000000000002036 (PMC11392101; doi:10.1097/JS9.0000000000002036)
Supplement: Supplementary file 3 [file js9-110-5704-s003.docx]

**Supplementary Table 1.** Baseline characteristics of participants stratified by varicose vein history after 1:3 propensity score matching

| Variable | Total | Varicose vein history | |  |
| --- | --- | --- | --- | --- |
|  |  | No | Yes | SMD |
| Number of participants (%) | 82944 | 62208 (75.0) | 20736 (25.0) |  |
| Age, years | 52.38±12.6 | 52.37±12.63 | 52.41±12.53 | 0.003 |
| Sex |  |  |  | 0.007 |
| Male | 26469 (31.9) | 19801 (31.8) | 6668 (32.2) |  |
| Female | 56475 (68.1) | 42407 (68.2) | 14068 (67.8) |  |
| Body mass index (kg/m^2^) | 23.77±3.20 | 23.79±3.21 | 23.73±3.16 | 0.019 |
| Household income |  |  |  | -0.005 |
| Q1, lowest | 16647 (20.1) | 12461 (20) | 4186 (20.2) |  |
| Q2 | 16014 (19.3) | 11997 (19.3) | 4017 (19.4) |  |
| Q3 | 20973 (25.3) | 15735 (25.3) | 5238 (25.3) |  |
| Q4, highest | 29310 (35.3) | 22015 (35.4) | 7295 (35.2) |  |
| Smoking status |  |  |  | 0.014 |
| Never | 63494 (76.6) | 47693 (76.7) | 15801 (76.2) |  |
| Former | 9724 (11.7) | 7295 (11.7) | 2429 (11.7) |  |
| Current | 9726 (11.7) | 7220 (11.6) | 2506 (12.1) |  |
| Alcohol consumption (days/week) |  |  |  | 0.007 |
| None | 54352 (65.5) | 40797 (65.6) | 13555 (65.4) |  |
| 1-2 | 20575 (24.8) | 15429 (24.8) | 5146 (24.8) |  |
| 3-4 | 5403 (6.5) | 4035 (6.5) | 1368 (6.6) |  |
| ≥ 5 | 2614 (3.2) | 1947 (3.1) | 667 (3.2) |  |
| Regular physical activity (days/week) |  |  |  | 0.010 |
| None | 52189 (62.9) | 39190 (63) | 12999 (62.7) |  |
| 1-2 | 16035 (19.3) | 12036 (19.4) | 3999 (19.3) |  |
| 3-4 | 8742 (10.5) | 6533 (10.5) | 2209 (10.7) |  |
| ≥ 5 | 5978 (7.2) | 4449 (7.2) | 1529 (7.4) |  |
| Comorbidities |  |  |  |  |
| Hypertension | 25171 (30.4) | 18889 (30.4) | 6282 (30.3) | 0.002 |
| Diabetes mellitus | 9420 (11.4) | 7005 (11.3) | 2415 (11.7) | 0.012 |
| Dyslipidemia | 21388 (25.8) | 16023 (25.8) | 5365 (25.9) | 0.003 |
| Chronic obstructive pulmonary disease | 7681 (9.3) | 5693 (9.2) | 1988 (9.6) | 0.015 |
| Liver disease | 7078 (8.5) | 5227 (8.4) | 1851 (8.9) | 0.019 |
| Renal disease | 1410 (1.7) | 809 (1.3) | 394 (1.9) | 0.008 |
| Stroke | 1593 (1.9) | 1143 (1.8) | 450 (2.2) | 0.024 |
| Myocardial infarction | 308 (0.4) | 218 (0.4) | 90 (0.4) | 0.009 |
| Cancer | 2230 (2.7) | 1624 (2.6) | 606 (2.9) | 0.019 |

*p-*value by Chi-square test. Data are expressed as the mean ± standard deviation, or n (%).

SMD, standardized mean difference. Q, quartile.

**Supplementary Table 2.** Association factors for the occurrence of atrial fibrillation in before PSM.

| Variable | Crude HR (95% CI) | p-value | Adjusted HR (95% CI) | p-value |
| --- | --- | --- | --- | --- |
| Age, years | 1.08 (1.07, 1.09) | <.001 | 1.07 (1.06, 1.08) | <.001 |
| Sex |  |  |  |  |
| Male | 1 (reference) |  | 1 (reference) |  |
| Female | 0.81 (0.80, 0.82) | <.001 | 0.68 (0.66, 0.69) | <.001 |
| Body mass index (kg/m^2^) | 1.06 (1.05, 1.07) | <.001 | 1.03 (1.02, 1.04) | <.001 |
| Household income |  |  |  |  |
| Q1, lowest | 1 (reference) |  | 1 (reference) |  |
| Q2 | 0.79 (0.77, 0.81) | <.001 | 0.97 (0.95, 0.99) | 0.007 |
| Q3 | 0.92 (0.9, 0.94) | <.001 | 0.98 (0.96, 1.00) | 0.094 |
| Q4, highest | 1.22 (1.19, 1.24) | <.001 | 0.97 (0.95, 0.99) | 0.005 |
| Smoking |  |  |  |  |
| Never | 1 (reference) |  | 1 (reference) |  |
| Former | 1.34 (1.32, 1.36) | <.001 | 1.04 (1.01, 1.06) | 0.002 |
| Current | 0.82 (0.80, 0.83) | <.001 | 1.08 (1.06, 1.11) | <.001 |
| Alcohol consumption (days/week) |  |  |  |  |
| None | 1 (reference) |  | 1 (reference) |  |
| 1-2 | 0.56 (0.55, 0.57) | <.001 | 0.98 (0.96, 0.99) | 0.009 |
| 3-4 | 0.83 (0.81, 0.85) | <.001 | 1.07 (1.04, 1.1) | <.001 |
| ≥5 | 1.52 (1.48, 1.56) | <.001 | 1.17 (1.13, 1.2) | <.001 |
| Regular physical activity (days/week) |  |  |  |  |
| None | 1 (reference) |  | 1 (reference) |  |
| 1-4 | 0.67 (0.66, 0.68) | <.001 | 0.93 (0.91, 0.95) | <.001 |
| 3-4 | 0.86 (0.84, 0.88) | <.001 | 0.95 (0.92, 0.97) | <.001 |
| ≥5 | 0.75 (0.65, 0.85) | <.001 | 0.95 (0.92, 0.97) | 0.001 |
| Comorbidities |  |  |  |  |
| Hypertension | 4.02 (3.96, 4.07) | <.001 | 1.51 (1.49, 1.54) | <.001 |
| Diabetes mellitus | 2.7 (2.66, 2.75) | <.001 | 1.07 (1.05, 1.09) | <.001 |
| Dyslipidemia | 2.33 (2.29, 2.36) | <.001 | 1.01 (0.99, 1.03) | 0.262 |
| Chronic obstructive pulmonary disease | 2.28 (2.23, 2.32) | <.001 | 1.21 (1.19, 1.24) | <.001 |
| Liver disease | 1.93 (1.89, 1.98) | <.001 | 1.12 (1.10, 1.15) | <.001 |
| Renal disease | 2.02 (1.53, 2.54) | <.001 | 1.32 (1.20, 1.46) | <.001 |
| Stroke | 3.99 (3.87, 4.12) | <.001 | 1.31 (1.27, 1.35) | <.001 |
| Myocardial infarction | 4.19 (3.88, 4.51) | <.001 | 1.48 (1.37, 1.60) | <.001 |
| Cancer | 2.03 (1.95, 2.12) | <.001 | 1.15 (1.10, 1.19) | <.001 |
| Varicose vein | 1.31 (1.22, 1.39) | <.001 | 1.13 (1.06, 1.21) | <.001 |

Multivariable model was adjusted with sex, age, body mass index, income levels, smoking, alcohol consumption, regular physical activity, hypertension, diabetes mellitus, dyslipidemia, chronic obstructive pulmonary disease, liver disease, renal disease, stroke, myocardial infarction, and cancer.

HR, hazard ratio; CI, confidence interval; Q, quartile.

**Supplementary Table 3.** Association factors for the occurrence of atrial fibrillation after PSM.

| Variable | Crude HR (95% CI) | p-value | Adjusted HR (95% CI) | p-value |
| --- | --- | --- | --- | --- |
| Age, years | 1.08 (1.08, 1.09) | <.001 | 1.07 (1.07, 1.08) | <.001 |
| Sex |  |  |  |  |
| Male | 1 (reference) |  | 1 (reference) |  |
| Female | 0.61 (0.56, 0.65) | <.0001 | 0.70 (0.64, 0.78) | <.001 |
| Body mass index (kg/m^2^) | 1.07 (1.06, 1.08) | <.0001 | 1.03 (1.02, 1.04) | <.001 |
| Household income |  |  |  |  |
| Q1, lowest | 1 (reference) |  | 1 (reference) |  |
| Q2 | 0.85 (0.75, 0.96) | 0.010 | 0.97 (0.85, 1.10) | 0.588 |
| Q3 | 1.08 (0.96, 1.20) | 0.198 | 1.06 (0.95, 1.19) | 0.290 |
| Q4, highest | 1.27 (1.14, 1.40) | <.001 | 1.01 (0.91, 1.12) | 0.863 |
| Smoking |  |  |  |  |
| Never | 1 (reference) |  | 1 (reference) |  |
| Former | 1.40 (1.27, 1.55) | <.001 | 0.93 (0.82, 1.06) | 0.269 |
| Current | 1.00 (0.89, 1.12) | 0.950 | 1.01 (0.86, 1.16) | 0.863 |
| Alcohol consumption (days/week) |  |  |  |  |
| None | 1 (reference) |  | 1 (reference) |  |
| 1-2 | 0.65 (0.59, 0.72) | <.001 | 0.96 (0.86, 1.07) | 0.484 |
| 3-4 | 1.15 (1.00, 1.32) | 0.043 | 1.21 (1.05, 1.41) | 0.011 |
| ≥5 | 1.59 (1.34, 1.88) | <.001 | 1.19 (0.99, 1.42) | 0.061 |
| Regular physical activity (days/week) |  |  |  |  |
| None | 1 (reference) |  | 1 (reference) |  |
| 1-4 | 0.69 (0.62, 0.77) | <.001 | 0.92 (0.83, 1.03) | 0.131 |
| 3-4 | 0.75 (0.65, 0.85) | <.001 | 0.82 (0.72, 0.94) | 0.004 |
| ≥5 | 0.89 (0.74, 1.05) | 0.273 | 0.92 (0.80, 1.05) | 0.209 |
| Comorbidities |  |  |  |  |
| Hypertension | 3.31 (3.07, 3.56) | <.001 | 1.50 (1.38, 1.63) | <.001 |
| Diabetes mellitus | 2.17 (1.98, 2.38) | <.001 | 1.06 (0.96, 1.17) | 0.240 |
| Dyslipidemia | 1.72 (1.59, 1.85) | <.001 | 0.93 (0.86, 1.01) | 0.101 |
| Chronic obstructive pulmonary disease | 1.83 (1.65, 2.03) | <.001 | 1.13 (1.02, 1.26) | 0.017 |
| Liver disease | 1.75 (1.57, 1.95) | <.001 | 1.23 (1.10, 1.38) | <.001 |
| Renal disease | 1.93 (1.74, 2.14) | <.001 | 1.35 (1.24, 1.45) | <.001 |
| Stroke | 3.99 (3.87, 4.12) | <.001 | 1.33 (1.13, 1.57) | <.001 |
| Myocardial infarction | 4.19 (3.88, 4.51) | <.001 | 1.75 (1.13, 2.69) | 0.011 |
| Cancer | 2.03 (1.95, 2.12) | <.001 | 0.99 (0.81, 1.21) | 0.925 |
| Varicose vein | 1.31 (1.22, 1.39) | <.001 | 1.17 (1.08, 1.27) | <.001 |

Multivariable model was adjusted with sex, age, BMI, income levels, smoking, alcohol consumption, regular physical activity, hypertension, diabetes mellitus, dyslipidemia, chronic obstructive pulmonary disease, liver disease, renal disease, stroke, myocardial infarction, and cancer.

HR, hazard ratio; CI, confidence interval; Q, quartile.

**Supplementary Table 4.** Subgroup analysis for association of varicose vein with incidence risk of atrial fibrillation

| Variable | Adjusted HR (95% CI) | p for interaction |
| --- | --- | --- |
| Age, <65 years | 1.02 (0.95 – 1.10) | 0.031 |
| Age, ≥65 years | 1.13 (1.04 – 1.23) |  |
| Male | 1.06 (0.93 – 1.20) | 0.726 |
| Female | 1.07 (0.95 – 1.28) |  |
| Body mass index <25 kg/m^2^ | 1.02 (1.01 – 1.04) | 0.284 |
| Body mass index ≥25 kg/m^2^ | 1.04 (1.02 – 1.06) |  |
| Household income Q1~Q3 | 0.97 (0.94 – 1.01) | 0.897 |
| Household income Q4 | 0.96 (0.94 – 1.01) |  |
| Smoking, never and former | 1.03 (1.01 – 1.05) | 0.579 |
| Smoking, current | 1.09 (1.06 – 1.12) |  |
| Alcohol consumption, none | 0.98 (0.94 – 1.02) | 0.103 |
| Alcohol consumption, one time or more/week | 1.01 (0.97 – 1.05) |  |
| Regular physical activity, none (days/week) | 1.01 (0.99 – 1.03) | 0.082 |
| Regular physical activity, one day or more/week | 0.94 (0.92 – 0.96) |  |
| Hypertension (-) | 1.37 (1.33 – 1.41) | 0.006 |
| Hypertension (+) | 1.68 (1.60 – 1.77) |  |
| Diabetes mellitus (-) | 1.06 (1.04 – 1.08) | 0.116 |
| Diabetes mellitus (+) | 1.08 (1.04 – 1.12) |  |
| Dyslipidemia (-) | 1.02 (0.98 – 1.06) | 0.673 |
| Dyslipidemia (+) | 1.01 (0.99 – 1.02) |  |
| Chronic obstructive pulmonary disease (-) | 1.19 (1.17 – 1.21) | 0.592 |
| Chronic obstructive pulmonary disease (+) | 1.22 (1.18 – 1.26) |  |
| Liver disease (-) | 1.10 (1.08 – 1.12) | 0.172 |
| Liver disease (+) | 1.14 (1.08 – 1.20) |  |
| Renal disease (-) | 1.29 (1.21 – 1.36) | 0.063 |
| Renal disease (+) | 1.34 (1.18 – 1.50) |  |
| Stroke (-) | 1.30 (1.25 - 1.35) | 0.284 |
| Stroke (+) | 1.33 (1.25 – 1.41) |  |
| Myocardial infarction (-) | 1.47 (1.37 - 1.57) | 0.316 |
| Myocardial infarction (+) | 1.49 (1.34 - 1.64) |  |
| Cancer (-) | 1.07 (1.02 - 1.12) | 0.402 |
| Cancer (+) | 1.16 (1.06 - 1.21) |  |

HR, hazard ratio; CI, confidence interval; Q, quartile.

**Supplementary Table 5.** Association factors for the occurrence of atrial fibrillation before and after PSM in population with ICD-10 code of I83.0-83.2.

| Variable | Before PSM  Adjusted HR (95% CI) | p-value | After PSM  Adjusted HR (95% CI) | p-value |
| --- | --- | --- | --- | --- |
| Age, years | 1.07 (1.06, 1.08) | <.001 | 1.07 (1.07, 1.08) | <.001 |
| Sex |  |  |  |  |
| Male | 1 (reference) |  | 1 (reference) |  |
| Female | 0.69 (0.67, 0.71) | <.001 | 0.71 (0.64, 0.78) | <.001 |
| Body mass index (kg/m^2^) | 1.03 (1.02, 1.04) | <.001 | 1.03 (1.02, 1.04) | <.001 |
| Household income |  |  |  |  |
| Q1, lowest | 1 (reference) |  | 1 (reference) |  |
| Q2 | 0.96 (0.94, 0.98) | 0.007 | 0.96 (0.85, 1.05) | 0.658 |
| Q3 | 0.98 (0.95, 1.00) | 0.099 | 0.97 (0.91, 1.03) | 0.473 |
| Q4, highest | 0.97 (0.94, 1.00) | 0.055 | 0.98 (0.92, 1.04) | 0.235 |
| Smoking |  |  |  |  |
| Never | 1 (reference) |  | 1 (reference) |  |
| Former | 1.04 (1.02, 1.06) | 0.003 | 1.03 (0.85, 1.21) | 0.792 |
| Current | 1.08 (1.05, 1.11) | <.001 | 1.05 (0.98, 1.11) | 0.821 |
| Alcohol consumption (days/week) |  |  |  |  |
| None | 1 (reference) |  | 1 (reference) |  |
| 1-2 | 0.96 (0.94, 0.98) | 0.001 | 0.96 (0.84, 1.08) | 0.386 |
| 3-4 | 1.08 (1.04, 1.12) | <.001 | 1.15 (1.02, 1.29) | 0.042 |
| ≥5 | 1.17 (1.13, 1.21) | <.001 | 1.18 (0.98, 1.37) | 0.063 |
| Regular physical activity (days/week) |  |  |  |  |
| None | 1 (reference) |  | 1 (reference) |  |
| 1-4 | 0.94 (0.92, 0.96) | <.001 | 0.91 (0.80, 1.02) | 0.791 |
| 3-4 | 0.95 (0.93, 0.97) | <.001 | 0.84 (0.73, 0.95) | 0.003 |
| ≥5 | 0.95 (0.93, 0.97) | 0.002 | 0.88 (0.64, 1.12) | 0.812 |
| Comorbidities |  |  |  |  |
| Hypertension | 1.54 (1.44, 1.65) | <.001 | 1.42 (1.31, 1.53) | <.001 |
| Diabetes mellitus | 1.09 (1.04, 1.14) | <.001 | 1.07 (0.92, 1.22) | 0.152 |
| Dyslipidemia | 1.04 (1.01, 1.07) | 0.002 | 1.02 (0.89, 1.15) | 0.562 |
| Chronic obstructive pulmonary disease | 1.24 (1.20, 1.28) | <.001 | 1.18 (1.11, 1.26) | 0.003 |
| Liver disease | 1.14 (1.10, 1.16) | <.001 | 1.11 (1.03, 1.19) | <.001 |
| Renal disease | 1.35 (1.21, 1.51) | <.001 | 1.21 (1.03, 1.40) | 0.021 |
| Stroke | 1.33 (1.25, 1.41) | <.001 | 1.15 (1.02, 1.27) | 0.006 |
| Myocardial infarction | 1.48 (1.38, 1.58) | <.001 | 1.41 (1.22, 1.62) | 0.003 |
| Cancer | 1.17 (1.11, 1.23) | <.001 | 1.06 (0.95, 1.17) | 0.417 |
| Varicose vein | 1.19 (1.09, 1.28) | 0.002 | 1.20 (1.10, 1.30) | 0.003 |

Multivariable model was adjusted with sex, age, BMI, income levels, smoking, alcohol consumption, regular physical activity, hypertension, diabetes mellitus, dyslipidemia, chronic obstructive pulmonary disease, liver disease, renal disease, stroke, myocardial infarction, and cancer.

HR, hazard ratio; CI, confidence interval; Q, quartile.

**Supplementary Table 6.** Association factors for the occurrence of atrial fibrillation after PSM in population with ICD-10 code of I83.9.

| Variable | Before PSM  Adjusted HR (95% CI) | p-value | After PSM  Adjusted HR (95% CI) | p-value |
| --- | --- | --- | --- | --- |
| Age, years | 1.07 (1.06, 1.08) | <.001 | 1.07 (1.07, 1.08) | <.001 |
| Sex |  |  |  |  |
| Male | 1 (reference) |  | 1 (reference) |  |
| Female | 0.69 (0.66, 0.72) | <.001 | 0.70 (0.65, 0.75) | <.001 |
| Body mass index (kg/m^2^) | 1.04 (1.02, 1.05) | <.001 | 1.03 (1.01, 1.05) | <.001 |
| Household income |  |  |  |  |
| Q1, lowest | 1 (reference) |  | 1 (reference) |  |
| Q2 | 0.95 (0.93, 0.98) | 0.007 | 0.95 (0.85, 1.05) | 0.642 |
| Q3 | 0.97 (0.94, 1.00) | 0.092 | 0.96 (0.89, 1.03) | 0.381 |
| Q4, highest | 0.96 (0.92, 1.00) | 0.089 | 0.96 (0.91, 1.01) | 0.752 |
| Smoking |  |  |  |  |
| Never | 1 (reference) |  | 1 (reference) |  |
| Former | 1.03 (1.02, 1.05) | 0.002 | 1.03 (1.01, 1.06) | 0.011 |
| Current | 1.07 (1.03, 1.11) | <.001 | 1.05 (1.03, 1.07) | <.001 |
| Alcohol consumption (days/week) |  |  |  |  |
| None | 1 (reference) |  | 1 (reference) |  |
| 1-2 | 0.97 (0.92, 1.02) | 0.462 | 0.95 (0.85, 1.05) | 0.295 |
| 3-4 | 1.11 (1.07, 1.15) | <.001 | 1.09 (1.01, 1.17) | 0.032 |
| ≥5 | 1.16 (1.13, 1.20) | <.001 | 1.14 (1.08, 1.20) | 0.018 |
| Regular physical activity (days/week) |  |  |  |  |
| None | 1 (reference) |  | 1 (reference) |  |
| 1-4 | 0.93 (0.91, 0.95) | <.001 | 0.94 (0.80, 1.08) | 0.632 |
| 3-4 | 0.94 (0.93, 0.95) | <.001 | 0.94 (0.73, 1.14) | 0.760 |
| ≥5 | 0.93 (0.90, 0.97) | <.001 | 0.92 (0.88, 0.96) | 0.024 |
| Comorbidities |  |  |  |  |
| Hypertension | 1.48 (1.45, 1.52) | <.001 | 1.41 (1.32, 1.50) | <.001 |
| Diabetes mellitus | 1.08 (1.05, 1.11) | <.001 | 1.07 (1.03, 1.11) | 0.021 |
| Dyslipidemia | 1.05 (1.02, 1.08) | 0.003 | 1.03 (0.95, 1.12) | 0.457 |
| Chronic obstructive pulmonary disease | 1.23 (1.18, 1.28) | <.001 | 1.19 (1.12, 1.26) | 0.004 |
| Liver disease | 1.15 (1.11, 1.19) | <.001 | 1.12 (1.04, 1.20) | <.001 |
| Renal disease | 1.31 (1.22, 1.40) | <.001 | 1.24 (1.13, 1.35) | 0.006 |
| Stroke | 1.21 (1.12, 1.31) | <.001 | 1.17 (1.03, 1.30) | 0.005 |
| Myocardial infarction | 1.34 (1.12, 1.57) | <.001 | 1.28 (1.13 1.43) | 0.002 |
| Cancer | 1.14 (1.10, 1.18) | <.001 | 1.08 (1.02, 1.14) | 0.007 |
| Varicose vein | 1.10 (1.04, 1.16) | 0.003 | 1.13 (1.03, 1.20) | <.001 |

Multivariable model was adjusted with sex, age, BMI, income levels, smoking, alcohol consumption, regular physical activity, hypertension, diabetes mellitus, dyslipidemia, chronic obstructive pulmonary disease, liver disease, renal disease, stroke, myocardial infarction, and cancer.

HR, hazard ratio; CI, confidence interval; Q, quartile.

**Supplementary Table 7.** Association of the presence of varicose vein with incidence risk of atrial fibrillation (landmark analysis)

|  | Number of participants | Number of events | Event rate (%) (95% CI) | Person-years | Incidence rate (per 1000 person-years) | Adjusted HR (95% CI) | p-value |
| --- | --- | --- | --- | --- | --- | --- | --- |
| Before-PSM  Presence of varicose vein history |  |  |  |  |  |  |  |
| (-) | 2656414 | 70720 | 2.66 (2.64, 2.68) | 25726452.89 | 2.75 | 1 (reference) |  |
| (+) I83.0, I83.1, I83.2, I83.9 | 24557 | 835 | 3.40 (3.17, 3.63) | 234765.90 | 3.51 | 1.13 (1.06, 1.21) | <0.001 |
| (+) I83.0, I83.1, I83.2 | 3684 | 142 | 3.85 (3.71, 3.99) | 36835.51 | 3.94 | 1.18 (1.10, 1.26) | 0.003 |
| (+) I83.9 | 20873 | 693 | 3.32 (3.01, 3.63) | 208734.53 | 3.42 | 1.10 (1.03, 1.17) | 0.002 |
| After-PSM |  |  |  |  |  |  |  |
| Presence of varicose vein history |  |  |  |  |  |  |  |
| (-) | 62208 | 1864 | 3.00 (2.86, 3.13) | 599772.53 | 3.11 | 1 (reference) |  |
| (+) I83.0, I83.1, I83.2, I83.9 | 20736 | 726 | 3.50 (3.25, 3.75) | 198260.13 | 3.66 | 1.17 (1.08, 1.27) | <0.001 |
| (+) I83.0, I83.1, I83.2 | 3110 | 123 | 3.97 (3.65, 4.29) | 31104.18 | 4.06 | 1.20 (1.12, 1.28) | 0.002 |
| (+) I83.9 | 17626 | 603 | 3.42 (3.13, 3.71) | 176256.35 | 3.51 | 1.12 (1.03, 1.21) | <0.001 |

Multivariable model was adjusted with age, sex, body mass index, income levels, smoking, alcohol consumption, regular physical activity, hypertension, diabetes mellitus, dyslipidemia, chronic obstructive pulmonary disease, liver disease, renal disease, stroke, myocardial infarction, and cancer.

CI, confidence interval; HR, hazard ratio; PSM, propensity score matching.

I83.0, I83.1, I83.2, I83.9: ICD-10 codes.

**Supplementary Table 8.** Association factors for the occurrence of atrial fibrillation before and after PSM in population with ICD-10 code of I83.0-83.2 (landmark analysis).

| Variable | Before PSM  Adjusted HR (95% CI) | p-value | After PSM  Adjusted HR (95% CI) | p-value |
| --- | --- | --- | --- | --- |
| Age, years | 1.07 (1.06, 1.08) | <.001 | 1.07 (1.06, 1.08) | <.001 |
| Sex |  |  |  |  |
| Male | 1 (reference) |  | 1 (reference) |  |
| Female | 0.70 (0.68, 0.72) | <.001 | 0.72 (0.66, 0.78) | <.001 |
| Body mass index (kg/m^2^) | 1.03 (1.02, 1.04) | <.001 | 1.03 (1.02, 1.04) | <.001 |
| Household income |  |  |  |  |
| Q1, lowest | 1 (reference) |  | 1 (reference) |  |
| Q2 | 0.95 (0.94, 0.96) | 0.001 | 0.96 (0.92, 1.01) | 0.103 |
| Q3 | 0.96 (0.91, 1.01) | 0.126 | 0.97 (0.92, 1.02) | 0.296 |
| Q4, highest | 0.97 (0.94, 1.00) | 0.058 | 0.97 (0.94, 1.00) | 0.053 |
| Smoking |  |  |  |  |
| Never | 1 (reference) |  | 1 (reference) |  |
| Former | 1.04 (1.01, 1.07) | 0.002 | 1.02 (0.84, 1.20) | 0.521 |
| Current | 1.07 (1.05, 1.09) | <.001 | 1.05 (0.97, 1.13) | 0.490 |
| Alcohol consumption (days/week) |  |  |  |  |
| None | 1 (reference) |  | 1 (reference) |  |
| 1-2 | 0.95 (0.90, 1.00) | 0.052 | 0.96 (0.86, 1.07) | 0.431 |
| 3-4 | 1.06 (0.98, 1.14) | 0.628 | 1.05 (0.97, 1.14) | 0.743 |
| ≥5 | 1.15 (1.10, 1.20) | <.001 | 1.12 (1.07, 1.17) | 0.032 |
| Regular physical activity (days/week) |  |  |  |  |
| None | 1 (reference) |  | 1 (reference) |  |
| 1-4 | 0.93 (0.91, 0.95) | <.001 | 0.94 (0.81, 1.07) | 0.254 |
| 3-4 | 0.95 (0.92, 0.98) | 0.004 | 0.91 (0.72, 1.11) | 0.611 |
| ≥5 | 0.96 (0.93, 0.99) | 0.003 | 0.89 (0.76, 1.02) | 0.292 |
| Comorbidities |  |  |  |  |
| Hypertension | 1.46 (1.33, 1.58) | <.001 | 1.45 (1.35, 1.56) | <.0001 |
| Diabetes mellitus | 1.11 (1.05, 1.17) | <.001 | 1.08 (1.02, 1.14) | 0.024 |
| Dyslipidemia | 1.03 (1.01, 1.05) | 0.003 | 1.02 (0.98, 1.01) | 0.092 |
| Chronic obstructive pulmonary disease | 1.18 (1.11, 1.25) | <.001 | 1.14 (1.07, 1.21) | 0.012 |
| Liver disease | 1.09 (1.06, 1.12) | <.001 | 1.11 (1.07, 1.14) | 0.003 |
| Renal disease | 1.28 (1.22, 1.34) | <.001 | 1.24 (1.15, 1.33) | 0.011 |
| Stroke | 1.21 (1.16, 1.26) | <.001 | 1.18 (1.12, 1.24) | 0.004 |
| Myocardial infarction | 1.36 (1.25, 1.48) | <.001 | 1.32 (1.12, 1.43) | 0.005 |
| Cancer | 1.15 (1.12, 1.18) | <.001 | 0.97 (0.82, 1.12) | 0.521 |
| Varicose vein | 1.18 (1.10, 1.26) | 0.003 | 1.20 (1.12, 1.28) | 0.002 |

Multivariable model was adjusted with sex, age, BMI, income levels, smoking, alcohol consumption, regular physical activity, hypertension, diabetes mellitus, dyslipidemia, chronic obstructive pulmonary disease, liver disease, renal disease, stroke, myocardial infarction, and cancer.

HR, hazard ratio; CI, confidence interval; Q, quartile.

**Supplementary Table 9.** Association factors for the occurrence of atrial fibrillation before and after PSM in population with ICD-10 code of I83.9 (landmark analysis).

| Variable | Before PSM  Adjusted HR (95% CI) | p-value | After PSM  Adjusted HR (95% CI) | p-value |
| --- | --- | --- | --- | --- |
| Age, years | 1.06 (1.05, 1.08) | <.001 | 1.07 (1.06, 1.08) | <.0001 |
| Sex |  |  |  |  |
| Male | 1 (reference) |  | 1 (reference) |  |
| Female | 0.69 (0.66, 0.72) | <.001 | 0.70 (0.62, 0.78) | <.0001 |
| Body mass index (kg/m^2^) | 1.03 (1.02, 1.04) | <.001 | 1.03 (1.02, 1.04) | <.0001 |
| Household income |  |  |  |  |
| Q1, lowest | 1 (reference) |  | 1 (reference) |  |
| Q2 | 0.96 (0.93, 0.99) | 0.003 | 0.96 (0.84, 1.08) | 0.316 |
| Q3 | 0.97 (0.94, 1.00) | 0.083 | 1.01 (0.94, 1.09) | 0.184 |
| Q4, highest | 0.96 (0.92, 1.00) | 0.065 | 1.02 (0.91, 1.12) | 0.741 |
| Smoking |  |  |  |  |
| Never | 1 (reference) |  | 1 (reference) |  |
| Former | 1.03 (1.01, 1.05) | 0.002 | 0.95 (0.84, 1.05) | 0.128 |
| Current | 1.07 (1.03, 1.11) | <.001 | 1.02 (0.87, 1.17) | 0.632 |
| Alcohol consumption (days/week) |  |  |  |  |
| None | 1 (reference) |  | 1 (reference) |  |
| 1-2 | 0.97 (0.93, 1.01) | 0.512 | 0.95 (0.89, 1.01) | 0.422 |
| 3-4 | 1.06 (1.03, 1.09) | <.001 | 1.07 (1.03, 1.11) | 0.015 |
| ≥5 | 1.15 (1.04, 1.26) | 0.004 | 1.14 (1.03, 1.25) | 0.002 |
| Regular physical activity (days/week) |  |  |  |  |
| None | 1 (reference) |  | 1 (reference) |  |
| 1-4 | 0.93 (0.88, 0.98) | 0.038 | 0.92 (0.81, 1.03) | 0.384 |
| 3-4 | 0.94 (0.90, 0.98) | 0.024 | 0.94 (0.83, 1.05) | 0.542 |
| ≥5 | 0.92 (0.90, 0.94) | 0.002 | 0.94 (0.84, 1.05) | 0.237 |
| Comorbidities |  |  |  |  |
| Hypertension | 1.48 (1.45, 1.51) | <.001 | 1.49 (1.46, 1.52) | <.0001 |
| Diabetes mellitus | 1.10 (1.05, 1.15) | <.001 | 1.07 (1.02, 1.12) | 0.002 |
| Dyslipidemia | 1.05 (1.02, 1.08) | 0.003 | 1.02 (0.86, 1.18) | 0.273 |
| Chronic obstructive pulmonary disease | 1.18 (1.15, 1.21) | <.001 | 1.16 (1.05, 1.27) | 0.006 |
| Liver disease | 1.13 (1.10, 1.16) | <.001 | 1.10 (1.06, 1.14) | 0.003 |
| Renal disease | 1.32 (1.20, 1.44) | <.001 | 1.30 (1.22, 1.38) | <.0001 |
| Stroke | 1.31 (1.24, 1.38) | <.001 | 1.28 (1.15, 1.41) | 0.006 |
| Myocardial infarction | 1.41 (1.33, 1.50) | 0.004 | 1.32 (1.02, 1.62) | 0.036 |
| Cancer | 1.14 (1.08, 1.20) | <.001 | 0.96 (0.85, 1.07) | 0.952 |
| Varicose vein | 1.10 (1.03, 1.17) | 0.002 | 1.12 (1.03, 1.21) | <0.001 |

Multivariable model was adjusted with sex, age, BMI, income levels, smoking, alcohol consumption, regular physical activity, hypertension, diabetes mellitus, dyslipidemia, chronic obstructive pulmonary disease, liver disease, renal disease, stroke, myocardial infarction, and cancer.

HR, hazard ratio; CI, confidence interval; Q, quartile.

**Supplementary Table 10.** Frequency of surgical treatment for varicose vein.

| Participants of surgical treatment for varicose vein, N=8,650 | | | | | | | | | | | |
| --- | --- | --- | --- | --- | --- | --- | --- | --- | --- | --- | --- |
| Number of treatments = 1 | | Number of treatments = 2 | | Number of treatments = 3 | | Number of treatments = 4 | | Number of treatments = 5 | | Number of treatments = 6 | |
| Procedure code | N (%) | Procedure code | N (%) | Procedure code | N (%) | Procedure code | N (%) | Procedure code | N (%) | Procedure code | N (%) |
| O0261 | 510 (5.90%) | O0215 & O0216 | 123 (1.42%) | O0215 & O0216 & O0217 | 26 (0.31%) | O0261 & O0263 & O0264 & O0265 | 4 (0.05%) | O0263 & O0264 & O0265 & O0266 & O2052 | 4(0.05%) | O0263 & O0265 & O0266 & O0267 & O0216 & O0217 | 4 (0.05%) |
| O0262 | 282 (3.26%) | O0215 & O0217 | 31 (0.36%) | O0261 & O0215 & O0216 | 4 (0.05%) | O0261 & O0263 & O0264 & O2052 | 4 (0.05%) |  |  |  |  |
| O0263 | 590 (6.82%) | O0216 & O0217 | 110 (1.27%) | O0261 & O0215 & O0217 | 4 (0.05%) | O0261 & O0263 & O0265 & O0216 | 9 (0.10%) |  |  |  |  |
| O0264 | 721 (8.34%) | O0261 & O0215 | 40 (0.46%) | O0261 & O0263 & O2052 | 4 (0.05%) | O0261 & O0264 & O0265 & O2052 | 4 (0.05%) |  |  |  |  |
| O0265 | 974 (11.26%) | O0261 & O0216 | 18 (0.20%) | O0261 & O0265 & O2052 | 4 (0.05%) | O0261 & O0265 & O0215 & O0217 | 4 (0.05%) |  |  |  |  |
| O0266 | 656 (7.58%) | O0261 & O0262 | 22 (0.25%) | O0261 & O2052 & O0215 | 9 (0.10%) | O0262 & O0264 & O0216 & O0217 | 4 (0.05%) |  |  |  |  |
| O0267 | 48 (0.56%) | O0261 & O0263 | 31 (0.36%) | O0262 & O0215 & O0216 | 4 (0.05%) | O0262 & O0264 & O0266 & O2052 | 4 (0.05%) |  |  |  |  |
| O2052 | 1214(14.04%) | O0261 & O0265 | 13 (0.15%) | O0262 & O0215 & O0217 | 4 (0.05%) | O0262 & O2052 & O0215 & O0216 | 4 (0.05%) |  |  |  |  |
| O0215 | 906 (10.48%) | O0261 & O0266 | 4 (0.05%) | O0262 & O0264 & O0265 | 9 (0.10%) | O0263 & O0264 & O0265 & O2052 | 4 (0.05%) |  |  |  |  |
| O0216 | 268 (3.10%) | O0261 & O0267 | 4 (0.05%) | O0262 & O0264 & O0266 | 4 (0.05%) | O0263 & O0265 & O0266 & O0217 | 4 (0.05%) |  |  |  |  |
| O0217 | 260 (3.00%) | O0261 & O2052 | 48 (0.56%) | O0262 & O0266 & O0217 | 4 (0.05%) | O0264 & O0265 & O2052 & O0217 | 4 (0.05%) |  |  |  |  |
|  |  | O0262 & O0215 | 15 (0.17%) | O0263 & O0215 & O0216 | 4 (0.05%) | O0264 & O0266 & O2052 & O0215 | 4 (0.05%) |  |  |  |  |
|  |  | O0262 & O0216 | 9 (0.10%) | O0263 & O0215 & O0217 | 4 (0.05%) | O0264 & O0266 & O2052 & O0216 | 4 (0.05%) |  |  |  |  |
|  |  | O0262 & O0217 | 9 (0.10%) | O0263 & O0264 & O0265 | 9 (0.10%) | O0265 & O0215 & O0216 & O0217 | 4 (0.05%) |  |  |  |  |
|  |  | O0262 & O0263 | 22 (0.25%) | O0263 & O0265 & O0216 | 4 (0.05%) |  |  |  |  |  |  |
|  |  | O0262 & O0264 | 18 (0.20%) | O0263 & O0265 & O0266 | 4 (0.05%) |  |  |  |  |  |  |
|  |  | O0262 & O0265 | 22 (0.25%) | O0263 & O0266 & O0216 | 4 (0.05%) |  |  |  |  |  |  |
|  |  | O0262 & O0266 | 9 (0.10%) | O0263 & O0266 & O0217 | 13 (0.15%) |  |  |  |  |  |  |
|  |  | O0262 & O2052 | 40 (0.46%) | O0263 & O2052 & O0215 | 4 (0.05%) |  |  |  |  |  |  |
|  |  | O0263 & O0215 | 13 (0.15%) | O0263 & O2052 & O0216 | 4 (0.05%) |  |  |  |  |  |  |
|  |  | O0263 & O0216 | 9 (0.10%) | O0264 & O0215 & O0216 | 13 (0.15%) |  |  |  |  |  |  |
|  |  | O0263 & O0217 | 40 (0.46%) | O0264 & O0216 & O0217 | 4 (0.05%) |  |  |  |  |  |  |
|  |  | O0263 & O0264 | 62 (0.71%) | O0264 & O0265 & O0215 | 9 (0.10%) |  |  |  |  |  |  |
|  |  | O0263 & O0265 | 53 (0.61%) | O0264 & O0265 & O0216 | 9 (0.10%) |  |  |  |  |  |  |
|  |  | O0263 & O0266 | 18 (0.20%) | O0264 & O0265 & O0266 | 13 (0.15%) |  |  |  |  |  |  |
|  |  | O0263 & O0267 | 4 (0.05%) | O0264 & O2052 & O0215 | 9 (0.10%) |  |  |  |  |  |  |
|  |  | O0263 & O2052 | 52 (0.6%) | O0264 & O2052 & O0217 | 4 (0.05%) |  |  |  |  |  |  |
|  |  | O0264 & O0215 | 40 (0.46%) | O0265 & O0215 & O0216 | 13 (0.15%) |  |  |  |  |  |  |
|  |  | O0264 & O0216 | 4 (0.05%) | O0265 & O0215 & O0217 | 4 (0.05%) |  |  |  |  |  |  |
|  |  | O0264 & O0217 | 48 (0.56%) | O0265 & O0216 & O0217 | 4 (0.05%) |  |  |  |  |  |  |
|  |  | O0264 & O0265 | 57 (0.66%) | O0265 & O0266 & O0215 | 4 (0.05%) |  |  |  |  |  |  |
|  |  | O0264 & O0266 | 114 (1.32%) | O0265 & O0266 & O0216 | 13 (0.15%) |  |  |  |  |  |  |
|  |  | O0264 & O2052 | 66 (0.76%) | O0265 & O0266 & O0217 | 13 (0.15%) |  |  |  |  |  |  |
|  |  | O0265 & O0215 | 53 (0.61%) | O0265 & O0266 & O2052 | 13 (0.15%) |  |  |  |  |  |  |
|  |  | O0265 & O0216 | 31 (0.36%) | O0265 & O0267 & O2052 | 4 (0.05%) |  |  |  |  |  |  |
|  |  | O0265 & O0217 | 44 (0.51%) | O0265 & O2052 & O0215 | 9 (0.10%) |  |  |  |  |  |  |
|  |  | O0265 & O0266 | 79 (0.92%) | O0265 & O2052 & O0216 | 4 (0.05%) |  |  |  |  |  |  |
|  |  | O0265 & O0267 | 4 (0.05%) | O0265 & O2052 & O0217 | 4 (0.05%) |  |  |  |  |  |  |
|  |  | O0265 & O2052 | 79 (0.92%) | O0266 & O0215 & O0216 | 4 (0.05%) |  |  |  |  |  |  |
|  |  | O0266 & O0215 | 79 (0.92%) | O0266 & O0215 & O0217 | 4 (0.05%) |  |  |  |  |  |  |
|  |  | O0266 & O0216 | 4 (0.05%) | O0266 & O0216 & O0217 | 4 (0.05%) |  |  |  |  |  |  |
|  |  | O0266 & O0217 | 79 (0.92%) | O0266 & O2052 & O0217 | 4 (0.05%) |  |  |  |  |  |  |
|  |  | O0266 & O2052 | 35 (0.41%) | O2052 & O0215 & O0216 | 11 (0.13%) |  |  |  |  |  |  |
|  |  | O0267 & O0215 | 4 (0.05%) | O2052 & O0216 & O0217 | 13 (0.15%) |  |  |  |  |  |  |
|  |  | O2052 & O0215 | 57 (0.66%) |  |  |  |  |  |  |  |  |
|  |  | O2052 & O0216 | 53 (0.61%) |  |  |  |  |  |  |  |  |
|  |  | O2052 & O0217 | 53 (0.61%) |  |  |  |  |  |  |  |  |

Abbreviations: N, number; The code names of surgical treatment corresponding to each code are as follows: Saphenous vein ligation & stab avulsion + perforator ligation(O0261); Saphenous vein ligation & stab avulsion – perforator ligation (O0262); Segmental stripping & stab avulsion + perforator ligation(O0263); Segmental stripping & stab avulsion – perforator ligation(O0264); Total stripping & stab avulsion + perforator ligation(O0265); Total stripping & stab avulsion – perforator ligation(O0266); Varicose vein operation, others(perineum)(O0267); Local resection(O2052); 1–3 sites(O0215); 4–6 sites (O0216); More than 7 sites(O0217).

**Supplementary Table 11.** Baseline characteristics of participants according to the performed surgical treatment for varicose vein.

| Variable | Total | Surgical treatment | | p-value |
| --- | --- | --- | --- | --- |
|  |  | No | Yes |  |
| Number of participants (%) | 24557 | 15907 (64.8) | 8650 (35.2) |  |
| Age, years | 52.40±12.56 | 52.53±13.28 | 52.15±11.12 | 0.024 |
| Sex |  |  |  | <.001 |
| Male | 7898 (32.2) | 4763 (29.9) | 3135 (36.2) |  |
| Female | 16659 (67.8) | 11144 (70.1) | 5515 (63.8) |  |
| Body mass index (kg/m^2^) | 23.74±3.18 | 23.70±3.24 | 23.83±3.06 | 0.002 |
| Household income |  |  |  | 0.754 |
| Q1, lowest | 4941 (20.1) | 3181 (20.0) | 1760 (20.4) |  |
| Q2 | 4789 (19.5) | 3114 (19.6) | 1675 (19.4) |  |
| Q3 | 6190 (25.2) | 4037 (25.4) | 2153 (24.9) |  |
| Q4, highest | 8637 (35.2) | 5575 (35.1) | 3062 (35.4) |  |
| Smoking status |  |  |  | <.001 |
| Never | 18725 (76.3) | 12378 (77.8) | 6347 (73.4) |  |
| Former | 2891 (11.8) | 1728 (10.9) | 1163 (13.5) |  |
| Current | 2941 (12.0) | 1801 (11.3) | 1140 (13.2) |  |
| Alcohol consumption (days/week) |  |  |  | <.001 |
| None | 16020 (65.2) | 10634 (66.9) | 5386 (62.3) |  |
| 1-2 | 6148 (25.0) | 3849 (24.2) | 2299 (26.6) |  |
| 3-4 | 1609 (6.6) | 963 (6.1) | 646 (7.5) |  |
| ≥ 5 | 780 (3.2) | 461 (2.9) | 319 (3.7) |  |
| Regular physical activity (days/week) |  |  |  | <.001 |
| None | 15388 (62.7) | 10202 (64.1) | 5186 (60.0) |  |
| 1-2 | 4730 (19.3) | 2980 (18.7) | 1750 (20.2) |  |
| 3-4 | 2614 (10.6) | 1605 (10.1) | 1009 (11.7) |  |
| ≥ 5 | 1825 (7.4) | 1120 (7.0) | 705 (8.2) |  |
| Comorbidities |  |  |  |  |
| Hypertension | 7443 (30.3) | 5000 (31.4) | 2443 (28.2) | <.001 |
| Diabetes mellitus | 2890 (11.8) | 2010 (12.6) | 880 (10.2) | <.001 |
| Dyslipidemia | 6358 (25.9) | 4254 (26.7) | 2104 (24.3) | <.001 |
| Chronic obstructive pulmonary disease | 2355 (9.6) | 1630 (10.3) | 725 (8.4) | <.001 |
| Liver disease | 2180 (8.9) | 1478 (9.3) | 702 (8.1) | 0.002 |
| Renal disease | 368 (1.5) | 223 (1.4) | 138 (1.6) | 0.007 |
| Stroke | 531 (2.2) | 385 (2.4) | 146 (1.7) | <.001 |
| Myocardial infarction | 53 (0.2) | 35 (0.2) | 18 (0.2) | 0.847 |
| Cancer | 708 (2.9) | 477 (3.0) | 231 (2.7) | 0.142 |

*p-*value by Chi-square test. Data are expressed as the mean ± standard deviation, or n (%).

Q, quartile.
